# Illiteracy and dementia

**DOI:** 10.1590/S1980-57642010DN40300002

**Published:** 2010

**Authors:** Sonia Maria Dozzi Brucki

**Affiliations:** MD, Behavioral and Cognitive Neurology Unit, Department of Neurology, University of São Paulo School of Medicine and Hospital Santa Marcelina, São Paulo SP, Brazil.

**Keywords:** dementia, illiteracy, cognition, education, neuropsychological evaluation

## Abstract

There is a current concept that illiteracy and lower educational levels are risk factors for cognitive decline and dementia. Our aims were to review the association between illiteracy and dementia; and to describe some results on neuropsychological findings in illiteracy. A literature search of the PubMed database was performed. The search terms were “dementia”, “illiteracy”, “neuropsychological evaluation”, “educational levels”, and “education”. Only papers published in Portuguese, English, and Spanish were reviewed. Illiteracy is an incontestable risk factor for dementia. It influences performance on almost cognitive tests. Many other factors could be connected to the high prevalence of dementia among illiterates: low cognitive reserve, poor control of cerebrovascular disease risk factors, difficulties in cognitive evaluation, and poor adaptation of neuropsychological tests for this specific population. Functional tests must be coupled with cognitive tests to ameliorate diagnostic accuracy.

## Abstract (pt)

Há um conceito corrente na literatura de que analfabetismo e baixos níveis educacionais são fatores de risco para declínio cognitivo e demência. Nossos objetivos foram revisar a associação entre analfabetismo e demência e descrever alguns resultados de achados neuropsicológicos no analfabetismo. A revisão de literatura foi realizada procurando-se os termos “dementia”, “illiteracy”, “neuropsychological evaluation”, “educational levels” e “education”. Somente artigos publicados em português, inglês e espanhol foram revistos. Analfabetismo é um fator de risco incontestável para demência, influenciando o desempenho em quase todos os testes. Muitos outros fatores podem ser ligados aos altos índices de demência entre analfabetos: baixa reserva cognitiva, controle pobre dos fatores de risco para doenças cerebrovasculares, dificuldades na avaliação cognitiva e adaptação pobre dos testes neuropsicológicos para este tipo de população. Testes funcionais devem ser acoplados aos testes cognitivos a fim de aumentar a acurácia diagnóstica.

The first neuropsychological on illiteracy studies were performed by Luria and Vygotsky in the 1930s during an expedition to Usbekistan in Central Asia, in a pioneering study in neuropsychology. These authors were interested in the extent to which reasoning, memory, and categorization are shaped by social and economic practices. This population worked in a traditional economy involving agriculture and farming, with many of them being illiterates or with one or two years of formal education. Luria and his researchers applied a number of cognitive tests, comparing illiterates who had never been to school with the educated individuals who had received a basic education. Some striking differences were identified on tasks of reasoning. Syllogisms were presented, first those containing practical experiences and second, those in which subjects were obligated to make inferences in a logical manner. For example: “*In the Far North, where there is snow, all bears are white. Novaya Zemlya is in the Far North. What colour are the bears there?*”. Some educated subjects refused to make inferences, declaring that they never had been in the North, and so could not answer the question. Uneducated participants displayed an empirical orientation, using their own experience to reject the premises. Another experiment was performed in women (more isolated, in part due to conservative teaching of Islam, with no social life) from remote tribes and women in teaching school (low educational level) and those who had no formal education, but with social activities. Men were divided into farming workers (illiterates) and workers of collective farms (with experience in production, distribution and administration, but little literacy) evaluated using the same geometric stimulus. Illiterates and isolated women named the stimulus by approximation with real objects (for example, tent for triangle or mirror for square), whereas in those with more education this type of naming was substituted for more abstract names.^1^ These findings were later replicated by Scribner and Cole (1981) with Vai tribes (in Liberia) in Western Africa. Illiterates made many errors on the reasoning problems but two years or more of schooling produced a dramatic improvement in accuracy.^2^

According to UNESCO, one in five adults in the world today is still not literate and two-thirds of these are women. Literacy remains a challenge for 759 million adults lacking minimum literacy skills. In Brazil, the illiteracy rate is 9.9% of the total population aged above 15 years (data from 2007). Although this rate is decreasing year by year, it is highest among elders, reaching 32.7% in rural environments (www.ibge.gov.br), but when divided according to age and environment (urban or rural), rates are very different (Figure 1). Besides having high rates of illiteracy in Brazil, there is a large number of individuals with less than one year of education (11.2% of people aged 15 years or more).

**Figure 1:**
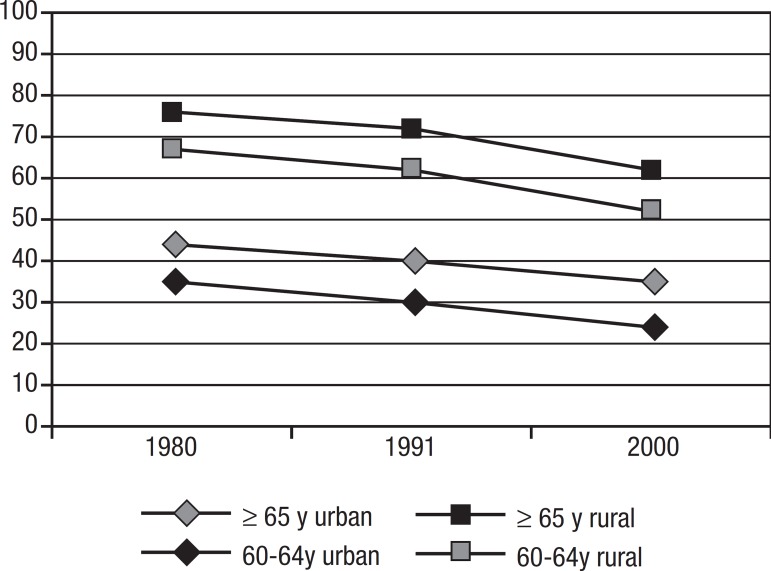
Illiteracy rates by age group, and urban or rural household.

The concern that low education is linked to greater prevalence of dementia has been discussed for many years. In 1987, the Shanghai survey demonstrated that lack of education was a major risk factor for and a major determinant of, the prevalence of dementia.^3^ In an earlier paper, Mortimer had stated that education would provide protection against dementia.^4^

Many studies have demonstrated that illiteracy and lower educational levels are risk factors for cognitive decline and dementia. In a pool of data from eight studies conducted in Latin America, illiteracy rate was 9.3% and the prevalence of dementia in illiterates was twice that of literates (Nitrini et al., 2009).^5^

Many factors have been correlated to higher prevalence of dementia among illiterates: low cognitive reserve, poor control of cerebrovascular disease risk factors, difficulties in cognitive evaluation, and poor adaptation of neuropsychological tests for this specific population.

References for this Review were identified through searches of PubMed with the search terms “dementia”, “illiteracy”, “neuropsychological evaluation”, “educational levels”, and “education”. Only papers published in Portuguese, English, and Spanish were reviewed. Abstracts were manually searched for relevance, and selected publications were searched for further relevant references.

## Epidemiology

Recently, pooled data from epidemiological surveys in six countries, on prevalence of dementia in Latin America, showed different rates among illiterates and literates of 15.67% and 7.16 %, respectively, with an overall rate of 7.1% of dementia in subjects aged 65 years or older.^5^ A survey conducted in Toledo (Spain) had found a similar rate of dementia in illiterates (13.3%), while another Spanish study in Murcia, demonstrated a greater risk of diagnosing amnestic mild cognitive impairment and dementia among illiterates.^6,7^ In a recent systematic review, the mean prevalence rates in elderly aged 65 years and older were 2.2% in Africa, 5.8% in Asia, 6.2% in North America, 7.1% in South America, and 8.9% in Europe.^8^ Heterogeneous rates across continents are evident, probably due to educational levels and different diagnostic criteria. Rodriguez et al. showed that prevalence of dementia can differ according to different criteria. Prevalence of dementia was underestimated when DSM-IV criterion were used compared to the 10/66 dementia algorithm, particularly in rural and less-developed settings.^9^ Dementia is unquestionably linked to illiteracy.^10-15^

A study comparing two populations of Monongahela Valley (USA) and a rural community (Ballabgarh) in India found that the incidence of dementia was lower in the latter sample, despite development of instruments suited to the population. The authors believed other confounders were present, such as cultural factors, diet and environmental exposures, and a low percentage of individuals living up to the age of risk prevented them from generalizing their findings.^16^ In a previous report, Salmon et al. pointed out that cultural differences can affect psychometric test performance, and hence, tests sensitive for dementia in one culture may not be so in another, especially if the cultures differ greatly in their level of education.^17^ Populations in the same country with the same spoken language were subject to cultural influences, as demonstrated by the authors with the Mini-Mental State examination, which revealed a difference between two samples from urban and rural areas.^18^

Other confounding factors when evaluating illiteracy rate in dementia include associations with income and socioeconomic factors, type of childhood development, and life expectancy, which could prevent people from surviving until old age. In addition, survivors may be protected from dementia by other factors such as genetic factors. One study in Brazil observed that illiteracy rate is strongly associated with life expectancy.^19^ In São Paulo (Brazil), dementia was more prevalent amongst participants who were illiterate, had non-skilled occupations and lower income. Illiteracy, poor occupational achievement and low income accounted for 22.0%, 38.5% and 38.5% of the cases of dementia, respectively.^20^

## Cognitive evaluation

Educated subjects outperformed illiterates on all cognitive measures. Tests to evaluate illiterates and subjects with low educational levels must be adapted and created especially for them. Many results have confirmed this issue.

Ardila et al. studied and compared illiterates with highly educated subjects, observed that all visuospatial tasks showed significant differences according to education. All measurements proved to be sensitive to the level of schooling on memory tasks, except for immediate recall of sentences.^21^ Another paper by the same authors, describes results on a neuropsychological examination of language and praxic abilities performed by 100 illiterates and 100 professionals. All the eight language subtests and seven praxic subtests disclosed statistically significant differences between educational levels. As described by researchers in language subtests, illiterates tended to omit commands. They presented difficulties in phonological abstraction, and paraphasias were observed in naming drawings. Illiterate subjects were unable to name the fingers, and phonological errors were observed in repetition of complex words. On praxic subtests, they showed loss of sequence and a tendency to use the hand as an instrument, and difficulties in representing sequences of movements without objects were found.^22^

Schooling seems to have a significant impact on mechanisms of cognition, and in this context illiteracy becomes far more than mere inability to read and write. Grossi et al. evaluated subjects in a rural village in Southern Italy, comparing illiterate elders to elders having little schooling (up to 3 years). The better educated subjects performed significantly better than illiterates on all measures (MMSE, Block Tapping Test, Verbal Memory Span, Long-Term Memory Test, History Recall, Constructional Apraxia Test and the Raven Matrices). The results were similar for reaction-time. Researchers believed that the primary notions learned during the first few years of schooling induce an improvement in mental strategies that is well preserved in the normal aging process.^23^ A study concerning literacy observed that scores on delayed recall, nonverbal abstraction, and category fluency were not influenced by literacy, in contrast to performance on naming, comprehension, verbal abstraction, orientation, and figure matching recognition. This study was able to distinguish the effects of literacy alone versus effects associated with formal education, since in addition to those who never learned to read and write, the study included subjects who were literate but had received little or no formal education.^24^

Illiterates utilized more semantic than phonological associations to retain memorization of words and provide names and verbal generation. Reis & Castro-Caldas evaluated subjects in a fishing community in the South of Portugal (with the same sociocultural backgrounds). They demonstrated that illiterates had difficulties in memorizing pairs of phonologically-related words compared to pairs of semantically-related words and were unable to generate words according to a formal criterion.^25^

Performance of illiterates and literates were different on two tests of long-term memory: delayed recall of a word list and a simple drawing list, with literates outperforming on the word list, whereas no differences were observed on delayed recall of drawings.^26^

Studies have demonstrated that visual and construction tasks are performed poorly by illiterates. Evaluating geometrical construction with sticks, Matute et al. observed that illiterates made more errors than semi-literates and literates, with lack of global fidelity to the model and committing of disarticulation errors.^27^

Reis et al. suggested that the visual system and/or visual interface and language system are organized differently in literates and illiterates. This was demonstrated by significantly better performance for naming two-dimensional representations of common objects by the literates. However, performance among illiterates and literates was the same when real objects were presented for naming. One reason is that lack of formal education implies that the illiterates rarely had the opportunity to systematically learn and practice the process of converting two dimensional forms into information, a skill that could be developed with reading and writing. A prolonged reaction time was observed for naming drawings and photographs of objects by illiterates, suggesting more difficulties in processing visual information.^28^ Brucki & Nitrini found a similar result, evaluating visual perception and motor speed using a cancellation task.^29^

In Brazil, many reports have described educational influence on neuropsychological measures. Screening tests, such as the Mini-Mental State Examination, are influenced by schooling, with different cut-off scores. Other cognitive batteries experienced the same effect, with significance difference among scores by literacy and educational level, observed on the Dementia Rating Scale,^30^ CERAD cognitive battery^31^ and ADAS-Cog.^32^ Surprisingly, although some tests are apparently free of schooling influence, such as Luria’s fist-edge-palm, tasks of immediate memory and naming simple drawings are affected by education.

Considering only one screening test, the Mini-Mental State Examination (MMSE), we observed a great influence of schooling, mainly on scores of illiterates. Many surveys have described lower and significantly different scores for illiterate groups.^33-40^

## Diagnose of dementia

It is essential to reach diagnose of cognitive dementia and functional impairment by comparing against the previous level of compromise in the patient. An early diagnosis is the fundamental key in clinical practice. It is necessary, besides an interview with patient and caregiver using cognitive and functional tests. A functional test is a good measure to be linked to cognitive test, such as the Functional Activities Questionnaire^41^ with another being the Informant Questionnaire on Cognitive Decline in the Elderly (IQCODE). Association of this kind of test to a screening test such as the MMSE improves diagnostic accuracy.^42-44^

## Comments

Clearly, substantial efforts are needed to maximize educational levels, as well as strict control of risk factors associated to vascular disease, in conjunction with improved socioeconomic conditions. In developing countries with heterogeneous education and socioeconomic levels, besides a task force to adapt cognitive tests to provide a better diagnosis and classification of subjects, functional measures of instrumental and daily living activities are required.
